# Complex Regional Pain Syndrome (CRPS/RSD) and Neuropathic Pain: Role of Intravenous Bisphosphonates as Analgesics

**DOI:** 10.1100/tsw.2008.33

**Published:** 2008-02-25

**Authors:** Jennifer Yanow, Marco Pappagallo, Letha Pillai

**Affiliations:** The Mount Sinai School of Medicine, Department of Anesthesiology, 5 East 98, Street, 6th fl., Box # 1192, New York, NY 10029, USA

**Keywords:** neuropathic pain, CRPS 1, CRPS 2, RSD, causalgia, bisphosphonates

## Abstract

Neuropathic pain is a sequela of dysfunction, injuries, or diseases of the peripheral and/or central nervous system pain pathways, which has historically been extremely difficult to treat. Complex regional pain syndrome (CRPS) types 1 and 2 are neuropathic pain conditions that have a long history in the medical literature but whose pathophysiology remains elusive and whose available treatment options remain few. While an exact animal model for CRPS doesn't yet exist, there are several animal models of neuropathic pain that develop behaviors of hypersensitivity, one of the hallmark signs of neuropathic pain in humans.

Bisphosphonates have been used for pathologic conditions associated with abnormal bone metabolism, such as osteoporosis, Paget’s disease and cancer-related bone pain for many years. More recently, results of clinical trials have indicated the potential role of bisphosphonates in the treatment of CRPS/RSD.

In this paper we will review the preclinical studies regarding the use of bisphosphonates as analgesics in animal models of neuropathic pain, and also summarize the clinical trials that have been done to date. We will give an overview of bisphosphonate pharmacology and discuss several potential mechanisms by which bisphosphonates may be analgesic in CRPS/RSD and bone pain of noncancer origin.

## NEUROPATHIC PAIN AND COMPLEX REGIONAL PAIN SYNDROME (CRPS/RSD)

Neuropathic pain is a sequela of dysfunction, injuries, or diseases of the peripheral (post-thoracotomy, HIV, diabetes) and/or central (stroke, multiple sclerosis, shingles) nervous system pain pathways. The pain signal is thought to be generated ectopically in small afferent fibers (A-delta, C-nerve fibers) and abnormally modulated by the central nervous system (CNS). Patients will exhibit several features classically used to describe neuropathic pain, such as hyperalgesia (exaggerated response to a mildly noxious stimulus) or allodynia (pain from non-noxious stimuli). Neuropathic pain continues beyond the time of normal tissue healing and is a considerable source of disability. Historically, it has been extremely difficult to treat and combinations of pharmacological agents, including antidepressants, anticonvulsants, and opioids, have often been used to alleviate suffering and pain in patients affected by neuropathic pain syndromes.

*Complex regional pain syndrome* (CRPS) *type 1*, formerly known as reflex sympathetic dystrophy (RSD), is a debilitating neuropathic pain disorder that has been recognized for more than a century. In 1993, the International Association for the Study of Pain (IASP) revised the IASP taxonomy by redefining and reclassifying RSD as CRPS type 1, and causalgia as CRPS type 2[49]. CRPS type 1 occurs following a period of immobilization or noxious event, and involves the development of continuing pain, allodynia, or hyperalgesia out of proportion to the initial event. CRPS type 2 occurs after a definable nerve injury, with the resultant pain, allodynia, or hyperalgesia not necessarily localized to the distribution of the injured nerve. Despite the long history of this disorder, the pathophysiology of CRPS/RSD has remained elusive. Of interest are the regional skeletal changes observed in several cases of CRPS/RSD. Although it has lost diagnostic purposes, a three-phase bone scintigraphy is still used in the evaluation of CRPS/RSD. Bone scintigraphy utilizes a technetium-99 radiolabeled bisphosphonate as an intravenous marker. The old RSD literature indicated a 50% diagnostic sensitivity and 90% diagnostic specificity for bone scintigraphy when performed in cases of RSD with duration of less than 6 months[1,2]. The typical RSD bone scan findings consisted of a homogeneous unilateral marker uptake (hyperperfusion) within the bone of the affected limb at both phase 1 (or "perfusion phase") occurring 30 sec after the marker injection and phase 2 (or "blood pool phase") occurring 2 min after the marker injection. During phase 3 (or "mineralization phase") at 3 h after injection, a characteristic uptake of the radiolabeled bisphosphonate is only observed in and around the joints of the affected limb. The significance of the bone scintigraphy findings in CRPS/RSD is unclear. Also undetermined is the potential role of bone pain mechanisms in CRPS/RSD. However, it is conceivable that a subgroup of patients with CRPS/RSD may suffer from a pathological pain state occurring in the bones of the symptomatic limb. For simplicity, we are going to use the term "neuropathic bone pain" to indicate this potential bone mechanism.

## PHYSIOLOGIC MECHANISMS OF "NEUROPATHIC BONE PAIN"

Immunohistochemical studies have revealed an extensive network of nerve fibers in the vicinity and within the skeleton[3]. Peptidergic sensory fibers, as well as sympathetic fibers, occur throughout the bone marrow, mineralized bone, and the periosteum. Both the periosteum and the bone marrow receive the highest degree of innervation. Multiple algogenic factors, such as a low pH, local synthesis of nerve growth factor (NGF), and release of proteases and inflammatory substances, such as cytokines and prostaglandins (PGs), might act synergistically to activate the extensive network of nociceptors innervating the periosteum, the cortical and trabecular bone, as well the bone marrow.

Activated osteoclasts produce an acidic microenvironment via the release of protons through vacuolar H+-ATPase[4,5]. A potential mechanism of bone pain may be the activation of two main groups of acid-sensing nociceptors[6]. Acid-sensing ion channels (ASICs) and the capsaicin receptor transient receptor potential vanilloid subtype 1 (TRVP1) are involved in proton-transduction mechanisms and in pain signal transmission[7,8,9]. ASIC-expressing nociceptors may also be involved in the transduction and transmission of mechanical pain[10]. It follows that some of the antinociceptive properties of agents that ameliorate bone pain, including bisphosphonates, may be attributed to inhibition of osteoclast activity and, in turn, to a decrease in proton concentration of the bone microenvironment.

NGF induces hyperalgesia by up-regulating the transcription for genes encoding pain receptors, such as capsaicin receptor or TRPV1, neuropeptides such as calcitonin gene-related peptide (CGRP), and the transmitter substance P. NGF was recently found to have a relevant role in bone pain. NGF-expressing cells and nociceptors with high-affinity tyrosine kinase receptors for NGF are found in bone[11,12,13]. NGF is produced by many cellular elements, including mast cells, macrophages, endothelial cells, osteocytes, osteoblasts, and bone marrow stromal cells[12,13], and its expression is enhanced in bone inflammation, bone cancer, trauma, and fractures. Therefore, inhibitors of osteoclasts or other cells that acidify the bone microenvironment or express NGF may prevent or reduce chronic bone pain. It is postulated that as neuropathic bone pain mechanisms are set in motion, pain becomes chronic and refractory to treatment.

There is evidence that tumor cells, endothelial cells, and activated macrophages, when directly exposed to bisphosphonates *in vitro* or *in vivo*, can suffer from bisphosphonate-induced toxic effects and even undergo apoptosis[14,15]. It is unknown, but possible, that the analgesic properties of bisphosphonates may not only be attributed to inhibition of osteoclast activity, but also to inhibition of NGF-expressing cells. Neuropeptides (CGRP and substance P) can activate mast cells (potent source of NGF) and endothelial cells to cause vasodilatation and plasma extravasation. Moreover, the presence of receptors for CGRP and substance P has recently been described on osteoclasts and osteoblasts. CGRP and substance P appear to regulate osteoclast formation, bone formation, and resorption[3].

It is hypothesized that bone pain mechanisms may have a more important clinical role than originally thought, and this may be relevant to subgroups of patients with chronic back pain and CRPS/RSD (Marco Pappagallo, oral communication, 2006).

## BISPHOSPHONATES: AN OVERVIEW

The pharmacological agents called bisphosphonates were initially developed as analogues of pyrophosphate, an agent commonly used as an antitartar agent in toothpaste. Traditionally, bisphosphonates have been used not only as analgesics for oncological bone pain, but also in the treatment of hypercalcemia of malignancy, osteolytic bone metastases, Paget's disease of bone, and osteoporosis. Earlier bisphosphonates, such as etidronate and clodronate, have been largely replaced by second-generation bisphosphonates, including pamidronate, as well as third-generation bisphosphonates, including zoledronic acid and ibandronate.

Bisphosphonates all have the P-C-P structure in common, which is similar to the P-O-P structure of native pyrophosphate. The bisphosphonates differ from one another at the two "R" groups. The ability of bisphosphonates to inhibit bone resorption depends on two specific properties of the bisphosphonate molecule. The two phosphonate groups plus the hydroxyl group at the R1 position result in high affinity for bone mineral, allowing efficient targeting of bisphosphonates to bone mineral surfaces. Once the bisphosphonate molecule is within the bone, the phosphorus groups and the R2 side chain determine the biological activity of the molecule and influence the ability of the drugs to interact with their targets.

The P-C-P moiety of the bisphosphonate molecule is responsible for its strong affinity for hydroxyapatite. The ability of the bisphosphonate to bind to hydroxyapatite crystals and to prevent growth/dissolution is enhanced when the R1 side chain is a hydroxyl group rather than a halogen atom. The P-C-P structure is also responsible for the ability of bisphosphonates to inhibit bone resorption. By altering the R2 side chain, more potent antiresorptive bisphosphonates (for example, pamidronate and alendronate) are produced. These bisphosphonates have an R2 side chain that contains a basic primary amino-nitrogen atom in an alkyl chain and are 10–100 times more potent than etidronate and clodronate. Compounds containing a tertiary amino-nitrogen, such as ibandronate, are even more potent, and those containing a nitrogen atom within a heterocyclic ring (risedronate, zoledronate) are up to 10,000 times more potent than etidronate[16].

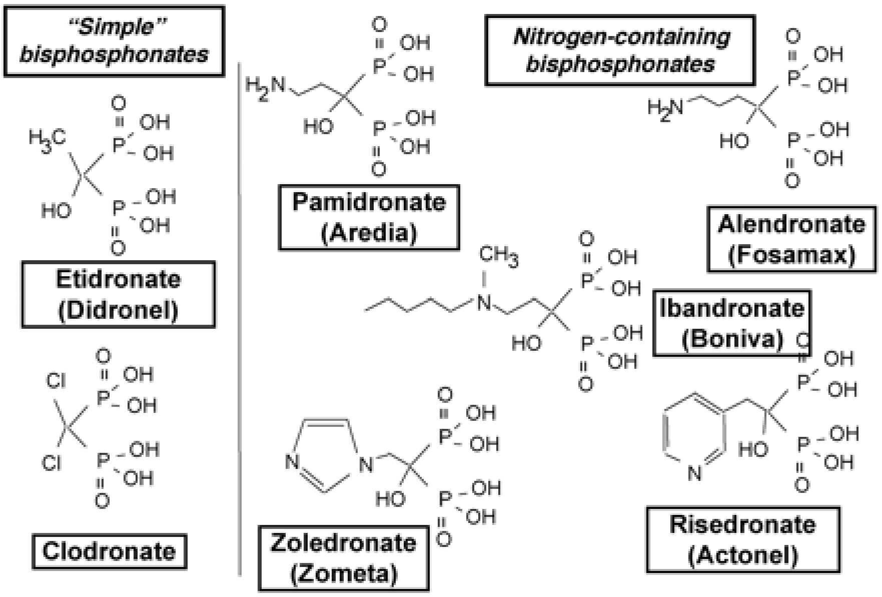

**FIGURE 1**. Chemical structure of the nitrogen-containing and non-nitrogen-containing bisphosphonates. Adapted from Russell, 2007[16].

Non-nitrogen-containing compounds, such as clodronate and etidronate, exert their effects by leading to the intracellular accumulation of metabolites that inhibit osteoclast function and cause their cell death. Nitrogen-containing bisphosphonates, such as alendronate, risedronate, and zoledronate, have bulkier side chains and are not metabolized. Instead, they interfere with metabolic reactions in the mevalonate biosynthetic pathway, protein prenylation, and ultimately the signaling functions of key regulatory proteins[16].

Bisphosphonates are available as oral or intravenous preparations. The major disadvantage of the orally utilized bisphosphonates is their extremely poor absorption in the gastrointestinal tract. Less than 1% of the oral dose is absorbed, and absorption is suppressed by food intake. In order to obtain a rapid and more considerable effect, a relatively high dose of bisphosphonates must be given by intravenous (IV) infusion. The pharmacokinetics of bisphosphonates is complex. These drugs remain in the bone, attached to hydroxyapatite crystals, for weeks to months.

Some of the bisphosphonates' biological effects are mediated through actions on the osteoclasts and likely on their cell precursors and other related cells, such as macrophages, CNS dendritic cells, and microglia. Bisphosphonates suppress osteoclast-mediated bone resorption via an intracellular effect on osteoclasts, and cause inhibition of osteoclast activity and reduction of the osteoclast life span. Of note, IV bisphosphonate treatment is associated with a rapid relief of bone pain[17,18], e.g., "immediately after the first infusion"[19], "within 10–14 days"[20], "within a matter of days"[21], and "within 1 month of infusion"[22].

When appropriately administrated, IV bisphosphonates are generally well tolerated and only associated with transient and manageable side effects (flu-like symptoms or acute phase reaction during the first 3 days following the infusion; symptoms usually respond to anti-inflammatory agents). Nevertheless, more recently and primarily in the oncology field, a serious condition known as osteonecrosis of the jaw (ONJ) has emerged as a potential treatment complication. ONJ may affect a subgroup of patients undergoing chronic IV bisphosphonate treatment for multiple myeloma, bone metastases from breast, prostate, or lung cancer. Some authors have reported a 5–10% incidence occurring after prolonged IV bisphosphonate treatment[25,26]. Tissue biopsies of the affected jaw lesions have revealed findings consistent with osteomyelitis[25]. Major risk factors include prolonged treatment with potent bisphosphonates (i.e., monthly IV administration for more than 1 year), poor oral hygiene, existing periodontal disease, periodontal surgery, dental implants, and a history of recent dental extractions.

There is a question about whether bisphosphonates inhibit fracture repair, although recent studies have shown no adverse bone healing in animals treated with ibandronate[26,27], as well as improved osseointegraion of metal implants[28].

## PRECLINICAL STUDIES: BISPHOSPHONATES IN NON-BONE-RELATED PAIN MODELS

Multiple lines of evidence gleaned from preclinical studies corroborate the analgesic action of bisphosphonates. Several preclinical studies revealed central and peripheral prolonged analgesic properties of the bisphosphonates for non-bone-related pain states[29,30]. These studies have also revealed a dose-dependent analgesic effect in animal models of both inflammatory and neuropathic pain[31,32,33,34].

There are several animal models of peripheral nerve injury and neuropathic pain produced by varying degrees of spinal nerve ligation, resulting in an incomplete denervation of the sciatic nerve territory through selective damage of certain contributing spinal nerves[35]. Three commonly used models are the Bennett model (loose ligation/chronic constriction of the entire sciatic nerve), the Chung model (tight ligation of an entire spinal segmental nerve close to the dorsal root ganglion), and the Seltzer model (tight ligation of a portion of the proximal sciatic nerve)[36]. These animals develop certain behaviors indicative of hypersensitivity, one of the hallmark signs of neuropathic pain in humans They display mechanical hypersensitivity to hair stimulation, mechanical pin-prick hyperalgesia, cold allodynia, and reduced latency to withdrawal in response to a heat stimulus; behaviors that persist for a prolonged period of time following the nerve ligation.

Walker et al. examined the analgesic efficacy of zoledronic acid in a rat model of neuropathic pain and mechanical hyperalgesia induced by partial ligation of the sciatic nerve. A reversal of mechanical hyperalgesia was observed 30 min following zoledronic acid administration[35]. Liu et al. investigated the effects of IV liposome-encapsulated clodronate in a rat model of neuropathic pain induced by sciatic nerve ligature. The authors showed that clodronate not only reduced the Wallerian nerve fiber degeneration and the recruitment of macrophages infiltrating the injured nerve, but also mechanical hyperalgesia[37]. Goicoechea et al. showed a dose- and time-dependent analgesic effect from intraperitoneal aledronate in the abdominal constriction test in mice[38]. Bonabello et al. showed both a peripherally and centrally mediated analgesic action of four bisphosphonates (clodronate, alendronate, pamidronate, and etidronate). The authors used two mouse pain models, the tail-flick test for thermal pain and the writhing test for chemical pain. The bisphosphonate effect was compared to those from morphine and acetylsalicylic acid. To study the peripheral effects of bisphosphonates, the medications were given intravenously. A dose-dependent antinociceptive effect was observed following administration of pamidronate, clodronate, and acetylsalicylic acid. Etidronate and alendronate also produced an analgesic effect, but only at the highest dose tested. To study the central effect of clodronate and pamidronate, the medications were administered intracerebroventricularly. Both bisphosphonates showed a dose-dependent antinociceptive effect[29].

## CLINICAL EXPERIENCE: BISPHOSPHONATES IN CRPS/RSD TRIALS

A review of the literature reveals multiple studies of bisphosphonates for CRPS/RSD. To date, four trials of intravenous pamidronate for CRPS/RSD have been published. In 1995, Maillefert et al. reported on seven of 11 patients with CRPS/RSD, who experienced clinically significant improvement from pamidronate therapy[39]. In 1997, Cortet et al. reported on 10 women and 13 men with CRPS/RSD, who showed highly significant pain reduction and physical functional improvement[40]. In 2001, Kubalek et al. treated 29 patients with CRPS/RSD[41]. Twenty-five of the patients experienced excellent pain relief from IV pamidronate at a dose of 60 mg/day for 3 consecutive days. Lastly, in a double-blind, randomized, placebo-controlled trial of IV pamidronate (n = 27), the active treatment group (n = 14) reported significant improvement in pain and physical function at 3 months postinfusion[42]. In a controlled trial of clodronate, 32 CRPS/RSD patients were randomized to receive either IV clodronate (300 mg daily) for 10 consecutive days or placebo[43]. This trial demonstrated significant efficacy of the clodronate treatment over placebo. Adami et al. reported that among 20 patients with CRPS/RSD, IV alendronate relieved pain by at least 50% in 13 patients, and those who received two infusions performed better than those who received one[44]. Finally, Manicourt et al. conducted a randomized controlled trial of oral alendronate (40 mg daily) for 8 weeks vs. placebo in patients (n = 40) with post-traumatic CRPS/RSD of the lower extremities[45]. The alendronate-treated patients exhibited a significant and sustained improvement in levels of spontaneous pain, mechanical pressure, and joint mobility. Several lines of evidence suggest that a subgroup of patients with CRPS/RSD might have bisphosphonate-responsive bone pain. Therefore, it is conceivable that bisphosphonate-responsive pain mechanisms might be involved in maintaining some of the symptoms of CRPS/RSD.

## CONCLUSION

The use of bisphosphonates for pathological conditions associated with abnormal bone metabolism, such as cancer bone pain, osteoporosis, and Paget’s disease, has been well documented. More recently, results of clinical trials have indicated the potential role of bisphosphonates in the treatment of the neuropathic pain syndrome known as CRPS/RSD. In this review, we have discussed several potential mechanisms by which bisphosphonates may be analgesic in CRPS/RSD and bone pain of noncancer origin. More research will be necessary to help elucidate these mechanisms. Some important information could be ascertained from studying the effects of IV bisphosphonates in patients with early CRPS/RSD. If the progression of disease was shown to be halted following therapy, in theory it may be possible for IV bisphosphonate treatment to lessen or even prevent the chronic neuropathic pain that eventually develops in CRPS/RSD.
